# Duration Judgments Over Multiple Elements

**DOI:** 10.3389/fpsyg.2012.00459

**Published:** 2012-11-05

**Authors:** Inci Ayhan, Yulia Revina, Aurelio Bruno, Alan Johnston

**Affiliations:** ^1^Cognitive, Perceptual and Brain Sciences, Division of Psychology and Language Sciences, University College LondonLondon, UK; ^2^CoMPLEX, University College LondonLondon, UK

**Keywords:** multiple timing, duration averaging, cueing paradigm

## Abstract

We investigated the limits of the number of events observers can simultaneously time. For single targets occurring in one of eight positions sensitivity to duration was improved for spatially pre-cued items as compared to post-cued items indicating that exogenous driven attention can improve duration discrimination. Sensitivity to duration for pre-cued items was also marginally better for single items as compared to eight items indicating that even after the allocation of focal attention, distractor items can interfere with the encoding of duration. For an eight item array discrimination was worse for post-cued locations as compared to pre-cued locations indicating both that attention can improve duration discrimination performance and that it was not possible to access a perfect memory trace of the duration of eight elements. The interference from the distractors in the pre-cued eight item array may reflect some mandatory averaging of target and distractor events. To further explore duration averaging we asked subjects to explicitly compare average durations of multiple item arrays against a single item standard duration. Duration discrimination thresholds were significantly lower for single elements as compared to multiple elements, showing that averaging, either automatically or intentionally, impairs duration discrimination. There was no set size effect. Performance was the same for averages of two and eight items, but performance with even an average of two items was worse than for one item. This was also true for sequential presentation indicating poor performance was not due to limits on the division of attention across items. Rather performance appears to be limited by an inability to remember or aggregate duration information from two or more items. Although it is possible to manipulate perceived duration locally, there appears to be no perceptual mechanisms for aggregating local durations across space.

## Introduction

Feedforward perceptual processes allow the visual brain to simultaneously register image motion or color across the visual field. Psychophysical tasks such as reporting which of two sine gratings drift faster, however, are not automatic and typically require the observer to set up a perceptual routine to make information which is implicit in a perceptual representation explicit. This is particularly true of temporal tasks (Johnston, 2010). For example, judging whether two events are simultaneous or not appears to require the individuation of the elements of the discrimination (Nishida and Johnston, 2002) and the bringing of that information together within a system that can reliably compare their timings. It is therefore important to make a distinction between the implicit representation of information on which temporal judgments are based and the more central attention demanding routines involved in temporal perception.

A recent line of research has shed light on temporal mechanisms underlying low-level implicit representations of relative durations in a visual array. Adaptation studies have shown that the perceived duration of brief intervals can be altered in specific spatial locations within the visual field by adaptation to flicker or motion (Johnston et al., 2006, 2008; Burr et al., 2007). Specifically, after adaptation to a 20-Hz drifting sine grating a 500-ms interval containing a 10-Hz drifting sine grating appears compressed by around 20%. This local manipulation of perceived time following temporal adaptation has been linked to adaptive changes in low-level mechanisms (Johnston et al., 2006, 2008; Ayhan et al., 2009) such as contrast gain control (Bruno and Johnston, 2010). These findings suggest that peripheral components of the perceptual system can be adapted in parallel influencing perceived duration, which is recovered later through some perceptual routine. Here we investigate the capacities of higher level perceptual routines involving attention and memory for duration encoding.

Duration judgment tasks are generally more demanding than psychophysical tasks requiring the judgment of other properties of an event (e.g., contrast or spatial length), since duration is not defined until the event is over. Searching for an odd duration item in a set of distractors cannot be done in parallel. Morgan et al. (2008) have shown that the precision with which subjects reported whether an “odd duration” was shorter or longer than the other elements of the display was affected by the set size. This search task result clearly shows that process of recovering duration is limited by a central attention demanding process.

Pylyshyn and Storm (1988) showed that observers can track up to four moving objects simultaneously, although Alvarez and Franconeri (2007) propose this is a processing rather than a structural limit and report that we can mentally track up to eight objects at optimal speeds. Our first question was whether this processing limit also applies to the encoding of the duration as well as the tracked position of multiple items. We discovered that observers found it very difficult to report the duration of a post-cued item in an array of eight items suggesting a difficulty in monitoring multiple items. To investigate the effectiveness of attention in selecting target items we compared performance in one item and eight item pre-cued arrays. We found some evidence of interference from distractors in the eight item array.

When local features are combined into a texture then subjects can make precise judgments about the mean values of the spatially distributed features (Parkes et al., 2001). Typically in perceptual discrimination judgments, such as judging the mean orientation of an array of line elements, discrimination thresholds decrease with increasing array size as the perceptual noise associated with the encoding of each element averages out (Parkes et al., 2001). If the duration of elements can be perceptually grouped we should expect duration discrimination to improve with set size. However, if each element is processed separately we might expect discrimination performance to decline with the number of elements contributing to the average due to increased cognitive load. Our second question therefore addressed whether observers can effectively average the durations of multiple items. To investigate the extent to which observers can combine durations we asked them to explicitly compare the average duration of a set of Gabor elements against a standard duration, for a range of array sizes. To evaluate whether this process is limited by the capacity to attend to multiple targets the intervals were presented sequentially as well as simultaneously. We found that judging the average duration of multiple events is a harder task than comparing the perceived durations of two single stimuli, ruling out perceptual averaging in the temporal domain. Set size and presentation type did not affect the precision with which the average duration of multiple events is judged. This indicated that encoding in this task was not attention limited and in fact observers found it difficult to integrate even just two separately presented durations.

## Materials and Methods

### Participants

Seven adults participated (three females, four males) in both experiments, five of which were naïve to the purpose of the experiment. All naïve subjects were undergraduate students at University College London (UCL). Visual acuity was normal or corrected-to-normal for all. The experiment was conducted in accordance with the ethical guidelines laid down by the UCL Division of Psychology and Language Sciences Ethics Committee.

### Apparatus

Observers were seated 57 cm from a 19″ Sony Trinitron Multiscan 500PS monitor, with a refresh rate of 100 Hz, driven by a VSG 2/5 visual stimulus generator (Cambridge Research Systems). The resolution of the monitor was 800 × 600 pixels. At this distance, the monitor subtended 40° × 30°. The head of the subject was restrained with a chinrest.

### Stimuli

Stimuli were horizontally oriented static Gabors – a Gabor being defined as a sinusoidal luminance grating (1 c/°) windowed by a Gaussian function. The Michelson contrast of the sine gratings was 100% and the SD of the Gaussian function was 1.25° of visual angle with each patch restricted to a 7.5° × 7.5°. In conditions where multiple stimuli were present during the comparison period, the Gabor patches were centered on an imaginary circle (diameter 17° of visual angle) around the center.

## Experiment 1

In a visual search paradigm, Morgan et al. (2008) have shown that recovering the duration of an odd item is limited by central attentional processes. However, to the best of our knowledge, no study has yet investigated how well observers can judge the combined duration of multiple elements. Here we studied multiple timing in a pre- and post-cue paradigm, where the pre-cue condition served to manipulate the amount of attention allocated to a single stimulus surrounded by multiple distractors and the post-cue condition reflected selection and recovery from working memory. We also compared the results to a control condition, where the test stimulus was presented with no distractor stimuli.

### Procedure

**Figure 1:**
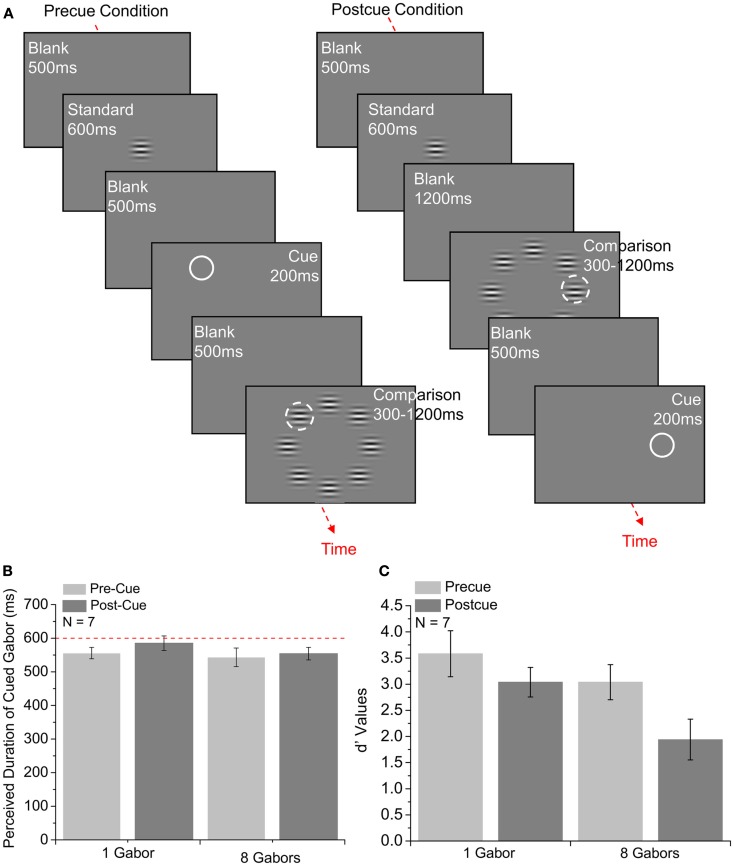
**The effect of cue type (pre- and post-cue) on the perceived duration and duration discrimination of multiple items**. The number of Gabor stimuli in the comparison array is shown beneath each condition in two graphs. **(A)** Time course of the binary choice experiments in which subjects made a duration judgment between a pre- or post-cued comparison Gabor stimulus on an imaginary circle against a standard presented centrally. **(B)** Mean perceived durations of cued Gabor for seven subjects are plotted as a function of the number of Gabor stimuli on the comparison array. The dashed line indicates the duration of the standard Gabor. Error bars show ±1 SE of the mean. Each point is derived from 210 to 350 trials. **(C)** Mean *d*′ values are plotted in the conditions shown in **(B)**. Error bars show ±1 SE of the mean. Each point is derived from 210 to 350 trials.

In Experiment 1 observers compared the duration of a comparison Gabor located on an imaginary circle against a standard presented centrally (Figure 1A). The comparison stimulus was embedded within an array of eight simultaneously presented Gabors (asynchronous onset: 0–200 ms). In two different conditions, the comparison was either pre- or post-cued (a white circle with inner and outer diameters of 6° and 6.25°). Pre-cuing was used to manipulate the amount of attention allocated to each location on the imaginary circle with multiple elements. The results were compared against a control condition in which the comparison was composed of just a single Gabor located in a random position on the imaginary circle. In this experiment, pre- and post-cue trials were blocked.

Whereas the standard Gabor had a fixed duration across trials (600 ms), the duration of the cued Gabor changed between 300 and 1200 ms to produce seven levels of comparison (300, 400, 500, 600, 800, 1000, or 1200 ms). The pseudo-logarithmic spacing was adopted as duration discrimination thresholds increase with duration according to a Weber law in the time range we used (for a review, see Grondin, 2001). The durations of the other Gabors were randomly taken from a normal distribution with mean of 600 ms and SD of 200 ms. Thus the average of the distractor Gabors was different in each trial and did not correlate with the duration of the cued Gabor. Calculating the average of the comparisons would have given no advantage in the task. The interstimulus interval between the standard and the comparison stimuli was kept at 1200 ms to balance the effects of memory in pre- and post-cue conditions. Observers were asked to report which of the two intervals was longer – a yes-no psychophysical paradigm. Responses were used to generate a psychometric function indicating the percent of trials in which the comparison was judged as longer than the standard for each subject. The 50% point on the psychometric function provided an estimate of the perceived duration of the standard. The slope of the psychometric function provides a measure of the discrimination threshold.

### Results

#### Perceived duration

Figure 1B shows the mean results for the perceived duration of the cued Gabor in single and eight-element-array conditions. Perceived duration values for the cued Gabor were calculated as the reverse of the expansion effect observed in the perceived duration of the standard stimulus (i.e., if the point of subjective equality was the standard duration plus 100 ms, this is plotted as the standard duration minus 100 ms – a compression effect of the cued comparison stimuli of 100 ms). A 2 × 2 (cue type × set size) repeated-measures ANOVA showed no significant difference between the mean perceived duration for the pre-cue (Mean = 549.64 ms, SE = 21.40) and post-cue (Mean = 569.70 ms, SE = 15.36) conditions, *F*(1, 6) = 4.078, *p *= 0.090. The main effect of set size was also not found to be statistically significant. There was no difference between the mean durations in the 1 Gabor (Mean = 570.47 ms, SE = 18.75) and 8 Gabor (Mean = 548.87 ms, SE = 21.16) conditions, *F*(1, 6) = 1.508, *p *> 0.1. Repeated-measures ANOVA indicated no interaction effect between the set size and the cue type, *F*(1, 6) < 1, *p* > 0.1. Since the main effects did not reach statistical significance, an average of the experimental conditions was computed (mean: 559.67 ms, SD = 47.49 ms) and compared against the duration of the standard stimulus (standard duration = 600 ms). Although overall the cued stimulus appeared reduced in duration, a one sample *t*-test showed the difference did not reach significance, *t*(6) = −2.247, *p *= 0.066. These results indicate that there was no substantive apparent expansion or compression of perceived duration for the cued stimuli, in either single versus multiple Gabor conditions, and for either cue type.

In this experiment, there were a small number of psychometric functions with shallow slopes for which performance did not reach the 84% point, therefore we also analyzed the data using signal detection methods. The PSEs (50% point) on these psychometric functions were well defined, however we also compute a signal detection measure of bias in responding. To obtain a model-independent measure of perceptual shifts, Criterion *C* values were computed using Palamedes version 1.3.1 data analysis toolbox (Prins and Kingdom, 2009). Those values were derived by signal detection analysis and were defined by the following formula:

(1) $$C=-\left[z\left(\text{pH}\right)+z\left(\text{pF}\right)\right]/2$$

where *z*(pH) and *z*(pF) are the *z*-values calculated for the proportion of hits and false alarms, respectively. Repeated-measures ANOVA on the *C* criterion values revealed main effects did not reach statistically significance for both the cue type, *F*(1, 6) = 4.719, *p* = 0.073, and the set size, *F*(1, 6) < 1, *p* > 0.1. The interaction effect between the cue type and the set size was also not found to be statistically significant, *F*(1, 6) < 1, *p* > 0.1, which together complement the results for the Gaussian integral model statistics. There was no measurable shift in the mean apparent duration or shift in criterion for the tested conditions.

#### Precision

Figure 1C shows the mean *d*′ values as a measure of precision with which participants made duration judgments in 1 and 8 Gabor pre- and post-cue conditions. Under the assumption that the stimuli are represented internally as random variables drawn from normal distributions, *d*′ gives a measure of the distance between the means of the distributions normalized to their standard variation:

(2) $$d^{\prime }=z\text{(pH)}-z\text{(pF)}$$

where *z*(pH) and *z*(pF) are the *z*-values calculated for the proportion of hits and false alarms, respectively. Larger *d*′ values indicate better discrimination performance, whereas lower *d*′ values indicate poorer precision.

A 2 × 2 repeated-measures ANOVA with the cue type and the set size as the two main factors revealed that the *d*′ values for the post-cue conditions (Mean = 2.488, SE = 0.315) were significantly lower than the *d*′ values for the pre-cue conditions (Mean = 3.218, SE = 0.360); *F*(1, 6) = 16.814, *p *= 0.006). This suggests that participants found the task easier in the pre-cue conditions. The difference between the *d*′ values for 8 Gabor conditions (Mean = 2.397, SE = 0.318), compared to 1 Gabor conditions (Mean = 3.310, SE = 0.359) were also found to be statistically significant, *F*(1, 6) = 24.344, *p *= 0.003, indicating a main set size effect – the precision with which subjects made duration judgments was higher in 1 Gabor conditions as compared to the 8 Gabor conditions. Repeated-measures ANOVA revealed no interaction effect between the cue type and the set size, *F*(1, 6) < 1, *p *> 0.1.

Following the ANOVA, paired-samples *t*-tests were carried out to examine individual contrast effects. The difference between 1 pre-cue (Mean = 3.582, SE = 0.44) and 1 post-cue conditions (Mean = 3.037, SE = 0.283) was statistically significant, *t*(6) = 2.986, *p *= 0.024), implying that the spatial uncertainty in the 1 Gabor post-cue condition decreased the precision of the duration judgments. Significant differences were also found between 1 post-cue (Mean = 3.037, SE = 0.283) and 8-post-cue (Mean = 1.939, SE = 0.391), *t*(6) = 4.186, *p *= 0.006) and 8 pre-cue (Mean = 2.854, SE = 0.335) and 8-post-cue (Mean = 1.939, SE = 0.391) conditions, *t*(6) = 2.589, *p *= 0.041 indicating that we do not encode and store duration at all locations. The difference between 1 pre-cue (Mean = 3.582, SE = 0.44) and 8 pre-cue (Mean = 2.854, SE = 0.335) conditions was marginally significant, *t*(6) = 2.366, *p *= 0.056, which suggest that the distractors interfered with the encoding of duration in the 8 Gabor pre-cue condition.

## Experiment 2

Experiment 1 showed that attention to pre-cued locations can significantly improve temporal discrimination, and that distractors can interfere with the precision with which subjects make duration judgments. The interference from the distractors in the pre-cued eight item array may reflect some mandatory averaging of target and distractor events. To further explore duration averaging, we carried out Experiment 2, in which participants made duration judgments on the average duration of an array of elements against a standard duration. To study how well attention can be divided across multiple elements, we manipulated array size, and the presentation mode (sequential versus simultaneous).

### Procedure

**Figure 2:**
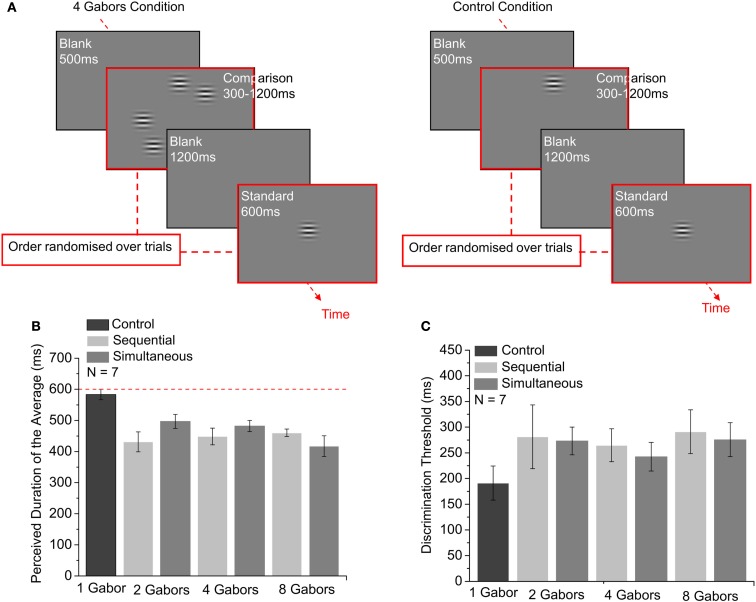
**The effect of the presentation type (sequential and simultaneous) and the set size (2, 4, and 8) on the precision and the perceived duration of the average of multiple items**. The number of Gabor stimuli on the comparison array is shown on the bottom of each condition in two graphs. **(A)** Time course of the binary choice experiments in which subjects made judgments on the average duration of a multiple array comparison stimulus on an imaginary circle against the duration of a standard presented centrally. **(B)** Mean perceived durations of the average are plotted as a function of the number of Gabor stimuli on the comparison array. The dashed line indicates the duration of the standard Gabor. Error bars show ±1 SE of the mean. Each point is derived from 140 trials. **(C)** Duration discrimination thresholds are plotted in the conditions shown in **(B)**. Error bars show ±1 SE of the mean. Each point is derived from 140 trials.

In Experiment 2, observers compared the average duration of a circular array of Gabor stimuli located around the center against a standard presented centrally (Figure 2A). The durations of the individual Gabors on the comparison array were determined in a pairwise fashion – the duration of the first element of a pair ranged between 110 and 1390 ms. The duration of the other element of the pair was set such that the average duration ranged between 300 and 1200 ms to produce seven levels of comparison (300, 400, 500, 600, 800, 1000, or 1200 ms) across trials with a condition that the minimum duration in the set could be no smaller than 110 ms. The order of presentation of the standard and comparison was randomized from trial to trial to control for time order effects (Jamieson and Petrusic, 1975).

In two different conditions, the comparison Gabors were presented simultaneously (onset asynchrony: 0–90 ms) or sequentially (20 ms interstimulus interval). The small onset asynchrony of the comparison stimuli was included to facilitate the segmentation and individuation of each element of the array. For each presentation condition (simultaneous and sequential), the number of elements in the comparison group was set to 2, 4, and 8 in blocked trials to study the effects of set size on the perceived average duration and duration discrimination threshold. The results were compared against a control condition in which the comparison was a single Gabor located at a random position on the imaginary circle. Responses were used to generate a psychometric function indicating the percentage of trials in which the comparison was judged as longer than the standard for each subject. The 50% point on the psychometric function provided an estimate of the perceived duration of the comparison Gabors. The discrimination threshold was defined as the SD of the Gaussian error distribution for the psychometric function, which corresponds to the difference in stimulus magnitude for the 84 and 50% points.

### Results

#### Perceived duration

Figure 2B shows how the presentation mode (sequential and simultaneous) and set size (2, 4, and 8 Gabors) affected the perceived average duration of an array of stimuli. As in Experiment 1, perceived average duration values were calculated as the reverse of expansion effect observed in the perceived duration of the standard stimulus stimulus (i.e., if the point of subjective equality was the standard duration plus 100 ms this is plotted as the standard duration minus 100 ms – a compression effect of the cued comparison stimuli of 100 ms). A 2 (presentation type) × 3 (set size) repeated-measures ANOVA indicated there was no significant difference in mean perceived duration in the sequential conditions (Mean = 446.36 ms, SE = 19.18), compared to the simultaneous conditions (Mean = 465.47 ms, S. E. = 18.38), *F*(1, 6) < 1, *p *> 0.1. There was also no significant difference between the mean perceived duration in the 2 Gabor (463.95 ms, SE = 12.89), 4 Gabor (465.11 ms, SE = 13.76), and 8 Gabor (438.684 ms, SE = 20.24) conditions, *F*(2, 12) = 1.772, *p *> 0.1. The interaction between the presentation mode and the set size was also not statistically significant, *F*(2, 12) = 2.57, *p *> 0.1, which together suggests that neither the presentation mode nor the set size (from two stimuli onward) has a statistically significant effect on the average perceived duration.

Since the presentation mode and the set size did not have a significant effect on the perceived average duration, the average of all the experimental conditions was computed and compared to the mean of the control condition. First, the mean of the control condition was compared against the duration of the standard stimulus (600 ms), which revealed no statistically significant difference, *t*(6) = −0.993, *p *> 0.1. A paired-samples *t*-test showed that the perceived duration for the experimental conditions (Mean = 455.91, SE = 13.08) is significantly different from the perceived duration in the control condition (Mean = 583.33, SE = 16.79), *t*(6) = 5.26, *p *= 0.002. This suggests that participants are accurate at comparing the duration of two stimuli, but the apparent duration of the average appears compressed when the number of stimuli is more than one and the task involved duration averaging. Thus the cognitive cost of averaging generates an apparent duration compression, but this cost does not increase with the number of elements, suggesting that cognitive load is already at ceiling when only two elements need to be integrated.

#### Precision

Figure 2C shows the duration discrimination threshold values as a measure of precision with which participants made duration judgments in the 1-, 2-, 4-, and 8-Gabor sequential and simultaneous presentation conditions. Discrimination thresholds are defined as the SD of the error distribution, which corresponds to the difference between the stimulus level for the 50 and 84% points of the psychometric function. Repeated-measures ANOVA with two levels of presentation mode (sequential and simultaneous) and three levels of set size (2, 4, and 8 Gabors; the control condition was excluded since it does not belong to either the sequential or simultaneous presentation mode) showed no significant differences in the discrimination thresholds for the sequential (278.99 ms, SE = 44.26) and the simultaneous presentation conditions [263.64 ms, SE = 27.07; *F*(1, 6) < 1, *p *> 0.1]. Differences in mean discrimination thresholds between 2 Gabor (277.10 ms, SE = 41.74), 4 Gabor (253.54 ms, SE = 25.17), and 8 Gabor conditions (283.31 ms, SE = 37.25) were also not found to be statistically significant, *F*(2, 12) = 1.283, *p *> 0.1, indicating no main effect for the set size. Finally, ANOVA showed no interaction effect between the presentation mode and the set size, *F*(2, 12) < 1, *p *> 0.1.

Since either of the main effects or the interaction were significantly different, data were averaged over the experimental conditions and compared to the control condition. The mean of the experimental conditions (Mean = 271.32 ms, SE = 33.56) was significantly higher than the control condition (Mean = 191.24 ms, SE = 33.22), *t*(6) = −7.75, *p *< 0.001, indicating that the participants were less precise in their duration judgments when there were more than one comparison stimulus and the task involved duration averaging. This indicates again that cognitive load was at ceiling when subjects were asked to average just two durations. Subjects could not take advantage of the potential reduction in perceptual error that can come from averaging multiple samples of the test interval.

## Discussion

We investigated the effect of attention- and working-memory-related limits on our ability to time multiple events. Our results indicate that:

1. Duration discrimination for a single target presented in one of eight positions is improved when the location of the target is spatially pre-cued, as the difference between *d*′ values in 1 pre-cue and 1 post-cue conditions in Experiment 1 was statistically significant (Figure 1C first and second columns).
2. Duration discrimination threshold for a spatially pre-cued element is marginally lower when presented alone as compared to when embedded in a multiple element array, as the difference between *d*′ values in 1 pre-cue and 8 pre-cue conditions in Experiment 1 was marginally significant (Figure 1C first and third columns).
3. Irrespective of the type of cueing, duration discrimination thresholds are lower for single targets as compared to targets presented with distractor items, as the *d*′ values for the post-cue conditions were significantly lower than the *d*′ values for the pre-cue conditions in Experiment 1 (Figure 1C).
4. Judging the average duration of multiple elements is harder than judging the duration of single items, as the difference between the *d*′ values for 8 Gabor conditions, compared to 1 Gabor conditions were found to be statistically significant (Figure 1C).
5. Duration discrimination performance in a duration averaging task is affected by neither the set size for set size greater than two (2, 4, and 8 elements) nor the presentation type (simultaneous and sequential), as in Experiment 2, no significant differences in the discrimination thresholds for the sequential and the simultaneous presentation conditions were found. Differences in mean discrimination thresholds between 2 Gabor, 4 Gabor, and 8 Gabor conditions were also not found to be statistically significant (Figure 2C).

In a visual search paradigm with multiple search items, Morgan et al. (2008) showed that the precision with which subjects report whether “the odd duration” was shorter or longer than the distractors was affected by the set size, indicating serial rather than parallel search for durations. Enhancement in duration discrimination for the spatially pre-cued targets in the single item conditions of Experiment 1 provides evidence that an attentional spatial focus can improve the timing of temporal events indicating a spatial component to event timing, which complements the observation that search for duration is serial rather than parallel.

Spotlight theories of attention propose that at a given point in time attentional selection is restricted to a single spatial location in the visual field and that this locus of attention is independent of eye movements (Posner, 1980; Eriksen and Yeh, 1985). Pylyshyn and Storm (1988) challenged the idea of single locus of attention, demonstrating that it is possible to track the spatial positions of four or five randomly moving identical items, providing evidence for divided spatial attention. Since the seminal study of Pylyshyn and Storm, a growing body of research has provided convergent evidence for multiple element tracking in the spatial domain (for a review, see Cavanagh and Alvarez, 2005). Although there exists a well-established consensus that observers can track the spatial position changes of multiple items, the number of objects that can be tracked simultaneously has been the subject of recent discussion. Alvarez and Franconeri (2007) have also shown that the limits on the performance in multiple tracking are not fixed but rather subject to available attentional resources and that the number of items subjects can track depends on the attentional demands of individual elements. In their study, Alvarez and Franconeri (2007) used speed to manipulate attentional demand. Their results demonstrated that the number of randomly moving objects subjects can track depends on the speed with which the tracked objects move and that given optimal speeds subjects can track simultaneously up to eight items. They also suggest that the more items to be tracked, the coarser the attentional window and the greater the likelihood that distractor items can interfere.

Here we addressed whether we can simultaneously time multiple items. Set size did not affect the discrimination performance of the subjects in our duration averaging task. Although observers were no worse with eight item displays than two item displays this cannot be interpreted as indicating parallel processing, as performance was worse when observers were required to compare average durations rather than single intervals against a standard duration. It would appear that subjects find it extremely difficult to average durations. In addition, the pattern of results was the same for averages of concurrent and sequential presentations. This indicates that performance was not limited by the capacity to divide processing resources between items. The fundamental problem appears to be an inability to successfully average the duration of intervals. This may be due to an inability to store durations, although this is required in two interval psychophysics paradigms, or that internal representation of duration information is not of a form that supports averaging.

When an ensemble of local stimulus features is processed as texture, the mean values of those spatially distributed features are extracted with high precision. Parkes et al. (2001) have shown that judgments of the orientation of a target suffer interference from surrounding distractors with different orientations. In addition, averaging introduces a reduction of mean orientation discrimination thresholds with set size. The increase in the discrimination thresholds for a task that involved explicit averaging of multiple durations compared to making judgments about two single intervals, suggests that unlike orientation signals, local temporal signals are not perceptually pooled across space, or grouped as a texture in the types of arrays used here.

Traditional models of time perception tend to dissociate temporal processing from spatial vision. Recent evidence (Johnston et al., 2006, 2008; Burr et al., 2007; Ayhan et al., 2009), however, has shown that adaptation to high temporal frequency induces spatially specific reductions in the apparent duration of sub-second intervals containing medium frequency drift or flicker. This indicates that there are peripheral neural mechanisms whose operation can be manipulated locally, however this does not imply the existence of autonomous clocks within early visual mechanisms. Rather, we propose that the process of timing visual events requires the establishment of a perceptual routine, which allows the duration of sensory events to be estimated. The establishment of this routine requires attention but once the event is individuated and while attention is maintained, perceptual mechanisms can be used for timing. These perceptual mechanisms can be manipulated through modality and spatially specific perceptual adaptation (Johnston et al., 2006).

We conclude that judging the durations of multiple events requires central attention demanding routines and that it is not possible to gain access to a perfect memory trace of the duration of multiple elements presented simultaneously. Averaging impairs duration discrimination irrespective of the number of elements, and duration discrimination thresholds for average intervals remain the same for both simultaneous and sequential presentations, implying that the poor performance is not due to limits on dividing attention across items.

## Conflict of Interest Statement

The authors declare that the research was conducted in the absence of any commercial or financial relationships that could be construed as a potential conflict of interest.
